# Learning from the private sector: towards a keener understanding of the end-user for microbicide introduction planning

**DOI:** 10.7448/IAS.17.3.19162

**Published:** 2014-09-08

**Authors:** Amy H Lin, Tiffany L Breger, Matthew Barnhart, Ann Kim, Charlotte Vangsgaard, Emily Harris

**Affiliations:** 1 United States Agency for International Development (USAID), Center for Accelerating Innovation and Impact Washington, DC, USA; 2 Department of Epidemiology, University of North Carolina Gillings School of Global Public Health Chapel Hill, NC, USA; 3 United States Agency for International Development (USAID), Office of HIV/AIDS Washington, DC, USA; 4 IDEO, Boston, MA, USA; 5 ReD Associates New York, NY, USA

**Keywords:** HIV, prevention, microbicides, end-user, user research, women, adherence, introduction planning

## Abstract

**Introduction:**

In planning for the introduction of vaginal microbicides and other new antiretroviral (ARV)-based prevention products for women, an in-depth understanding of potential end-users will be critically important to inform strategies to optimize uptake and long-term adherence. User-centred private sector companies have contributed to the successful launch of many different types of products, employing methods drawn from behavioural and social sciences to shape product designs, marketing messages and communication channels. Examples of how the private sector has adapted and applied these techniques to make decisions around product messaging and targeting may be instructive for adaptation to microbicide introduction.

**Discussion:**

In preparing to introduce a product, user-centred private sector companies employ diverse methods to understand the target population and their lifestyles, values and motivations. ReD Associates’ observational research on user behaviours in the packaged food and diabetes fields illustrates how ‘tag along’ or ‘shadowing’ techniques can identify sources of non-adherence. Another open-ended method is self-documentation, and IDEO's mammography research utilized this to uncover user motivations that extended beyond health. Mapping the user journey is a quantitative approach for outlining critical decision-making stages, and Monitor Inclusive Markets applied this framework to identify toilet design opportunities for the rural poor. Through an iterative process, these various techniques can generate hypotheses on user drop-off points, quantify where drop-off is highest and prioritize areas of further research to uncover usage barriers. Although research constraints exist, these types of user-centred techniques have helped create effective messaging, product positioning and packaging of health products as well as family planning information. These methods can be applied to microbicide acceptability testing outside of clinical trials to design microbicide marketing that enhances product usage.

**Conclusions:**

The introduction of microbicide products presents an ideal opportunity to draw on the insights from user-centred private sector companies’ approaches, which can complement other methods that have been more commonly utilized in microbicide research to date. As microbicides move from clinical trials to real-world implementation, there will be more opportunities to combine a variety of approaches to understand end-users, which can lead to a more effective product launch and ultimately greater impact on preventing HIV infections.

## Introduction

With several ground-breaking possibilities for women-initiated methods of HIV prevention on the horizon, it is critical to begin planning for product introduction. Recent trials of antiretroviral (ARV)-based prevention products have demonstrated that pre-exposure prophylaxis (PrEP) will be effective if adherence is sufficient [1–5]. Though a lack of efficacy was observed in the FEM-PrEP [6] and VOICE (7) trials of PrEP among young women in sub-Saharan Africa, product use was low as measured by drug levels. Thus, hopes remain high that trials of either dapivirine ring (in the Ring Study and ASPIRE) and/or tenofovir gel (in FACTS 001) will confirm the efficacy observed in CAPRISA 004 and ultimately lead to licensure and introduction of one or more new vaginally administered products for HIV prevention.

The importance of integrating behavioural and social sciences in biomedical microbicide trials is widely recognized [6, 8–12], and behavioural science methods have been emphasized in past and on-going HIV research and intervention methodology. While it is beyond the scope of this commentary to provide a comprehensive review of all literature to date, the diversity in the quantitative and qualitative behavioural science methods used to support microbicide planning efforts is noteworthy. Previous and on-going microbicide research has included discrete choice experiments exploring the effect of efficacy and contraceptive potential on a woman's stated willingness to use microbicides [13]; focus groups, interviews and questionnaires examining microbicide acceptability [14–20]; analysis of disclosure of trial participation on microbicide use and adherence [21]; and Motivational Interviewing to identify and address trial participants’ barriers to adherence [22]. However, although behavioural science methods have been used in smaller studies of non-trial participants to examine factors such as perceptions of topical vaginal gels [23, 24] and intravaginal insertion practices [25], studies with this broader community have not been the norm, which has limited data related to potential microbicide end-users. Furthermore, methods from the broader social sciences have not been frequently utilized in HIV research and intervention methodology with either trial or non-trial participants.

As microbicide product development moves from clinical trials to real-world implementation in the coming years, there will be more opportunities to use complementary methods to understand women's interaction with a real product in a less artificial context. Deepening awareness of the barriers to microbicide use and access in a more demographically diverse population will be essential to ensuring this prevention method meets its potential to empower women in their sexual relationships and health. In this regard, ‘user-centred’ private sector companies (distinguished from marketing firms by a more intensive and open-ended approach to understand the end-user) have demonstrated success in increasing uptake of many different types of products. This has been achieved through utilizing a wide range of techniques to understand the target population as well as their lifestyles and broader environment [23, 26–33]. While behavioural and social sciences provide the foundation for this approach, examples of how the private sector has applied these techniques to make decisions around product messaging and targeting for effective product introduction can be instructive for the public sector's adaptation to the microbicide arena, particularly in the post-licensure phase of microbicide research.

## Discussion

To understand the underlying themes relevant to a new product, user-centred private sector design and marketing firms typically begin by speaking to and studying a wide range of individuals – not only potential end-users outside of the clinical trial context but also other individuals in the environment who may have widely disparate views [28, 32]. Speaking to ‘extreme’ users (e.g. those who embrace abstinence, as well as those who prefer sex without any protection) uncovers attitudes and beliefs that inform the understanding of the target user [32]. While prevention of HIV is the main objective for the public health world, for the user, that may not be the only mental framework. In the case of microbicides, perceptions of the product and HIV, as well as larger issues like beauty, wellness and social norms must be considered for a successful rollout.

Moreover, to fully understand user experiences, perceptions and underlying motivations, the private sector focuses on the broader context affecting decisions that are made around product use, investing heavily in understanding the larger ecosystem of values, beliefs, lifestyles, habits and other influencers of behaviour [27, 28, 33]. While methods used in microbicide trials such as in-depth interviews, focus groups and surveys are important for understanding end-users, one potential limitation is that they can produce an artificial situation where respondents answer according to what they believe is expected and may not reveal what they actually think or do [34]. The flexibility and open-ended nature of in-context observation can potentially mitigate this social desirability bias since this approach does not rely entirely on the interviewee's accountability and interest in articulating underlying motivations [33]. Additionally, it can help generate new hypotheses since questions are not pre-determined and will organically arise from real-time observations [26, 28].

### In-depth observational methods to understand end-users

One method of understanding a user's context and lifestyle is to follow users through their habits and routines, referred to as ‘tag along’ or ‘shadowing’ [28]. This technique can be used to complement in-depth interviews by revealing disconnects between individuals’ aspirations versus actions. ReD Associates demonstrated this in their work in packaged food (ReD Associates is an innovation and strategy consultancy that employs the methods of social science and market analysis). When interviewing mothers about how they choose between different types of food, researchers heard an emphasis on nutrition, organic ingredients and other aspects that emphasized health but found packaged foods that were primarily convenient, fast and easy to use upon looking in the refrigerator. Because similar discrepancies have been recognized between self-reported and measured microbicide adherence [6], these observational techniques might provide an additional source of information from lifestyles or emotional cues to triangulate and better interpret self-reported data.

Furthermore, these techniques have been useful in revealing the role of stigma and other root causes of behaviours. In the diabetes field, ReD Associates’ observational research found that patients would skip shots when in public due to a sense of self-consciousness or a fear of stigma from being identified as diabetic, contradicting the industry's prior assumption that lack of consistency was due to forgetfulness. This insight emphasized the need for discretion – for example, through less intrusive product designs with syringes that looked more like pens – over an intervention based on setting up timed or automated reminders.

While it is understood generally that HIV-related stigma affects microbicide use, our understanding of more specific facilitators of, and barriers to, microbicide use might be enhanced by direct observational research. Though it would not be possible for a researcher to tag along in every situation, and obviously not during sexual intercourse, a researcher may still gain valuable insight by joining a woman as she starts her weekend to observe her social interactions, the physical environment of her home and the places she goes, and even what she carries in her handbag to glean what she associates with sex. Post-observation, the researcher can follow-up with women to determine what sexual health choices were made. This can provide insight into aspects of daily life routines that influence usage of and adherence to microbicides. For example, women might associate cosmetics with beauty and microbicides with medication, which could result in bringing cosmetics – but not a microbicide – to a bar or to meet a sexual partner. This type of observation and usage driver can inform microbicide design so that packaging and marketing invoke the branding of beauty products instead of medications.

Another observational technique commonly used by the user-centred private sector is self-documentation, or enabling users to create photo and video diaries [32]. IDEO recently engaged in a project to uncover women's attitudes and experiences around mammography in the United States (IDEO is an international design and consultancy firm). Rather than accompanying each woman to individual mammogram appointments, the women were given video cameras and recorded their reactions and thoughts before, during and after the mammogram appointment. Giving women the freedom to record in the intimacy of their homes, doctor's offices and other relevant settings enabled open and comfortable reflection, revealing that some women were sceptical of mammograms, while others experienced mammograms as life-saving. Participation in the exercise over time highlighted how reasons for adherence to mammograms extended beyond cancer detection to larger themes around femininity, wellness, self-confidence and modelling behaviour for daughters.

Given the private nature of microbicide use, application of this technique can increase users’ sense of agency in providing feedback. The approach addresses practical needs (since it is not feasible for researchers to ‘embed’ themselves in women's sexual lives), while giving users flexibility and room for privacy. Mobile phones with photo and video capability are becoming more common in developing country settings and might be viewed by microbicide users as an interface that is more approachable than options such as writing essays or completing written questionnaires – and less subject to social desirability bias than in-person interviews. Although this might not be possible in all contexts due to privacy or institutional review board concerns, technology is becoming further integrated into research practice, and similar methods such as PhotoVoice, are also popular in advocacy efforts and women's health [35, 36]. Photo and video journaling can provide insight into the subject of microbicides in users’ minds, the connection to themes beyond the direct purpose of HIV prevention and the interlinked associations that may influence product uptake and usage.

### User journey mapping

Mapping the user journey is another user-centred private sector approach that goes hand-in-hand with observational research [37] and can be employed during the post-licensure phase when microbicides are introduced. This quantitative approach provides a framework for outlining the critical decision-making stages a potential user passes through in order to become a regular product user, allowing researchers to better understand potential drop-off points and calculate attrition rates for each stage. Illustrating this structure is part of an iterative process of end-user research in which initial observational research can generate hypotheses regarding potential drop-off points, and user journey mapping can calculate where drop-off is highest. This can then inform areas where more in-depth observational research can most efficiently and effectively hone in on root causes for these drop-offs.

Monitor Inclusive Markets (MIM) created this type of decision-making map through its customer research in Bihar, India, around willingness of the rural poor to purchase toilets (MIM is a unit of the Monitor group that focuses on market-based solutions for social change). Mapped against the user journey, this research revealed that financial constraints were substantial barriers at the purchase stage, and residents who may otherwise have bought a toilet found it unaffordable. Upon further investigation of this stage, researchers discovered that potential users wanted a long-term sanitation solution and assumed that requiring a 10-foot deep waste pit posed a significant cost. Armed with this knowledge, researchers were able to suggest an alternative, long-term toilet design with lower upfront cost, by using two five-foot deep pits [38]. The user journey analysis also revealed that key influencers of target users were friends, community members and local masons, suggesting the importance of building support for this new design among these trusted information sources.

**Figure 1:**
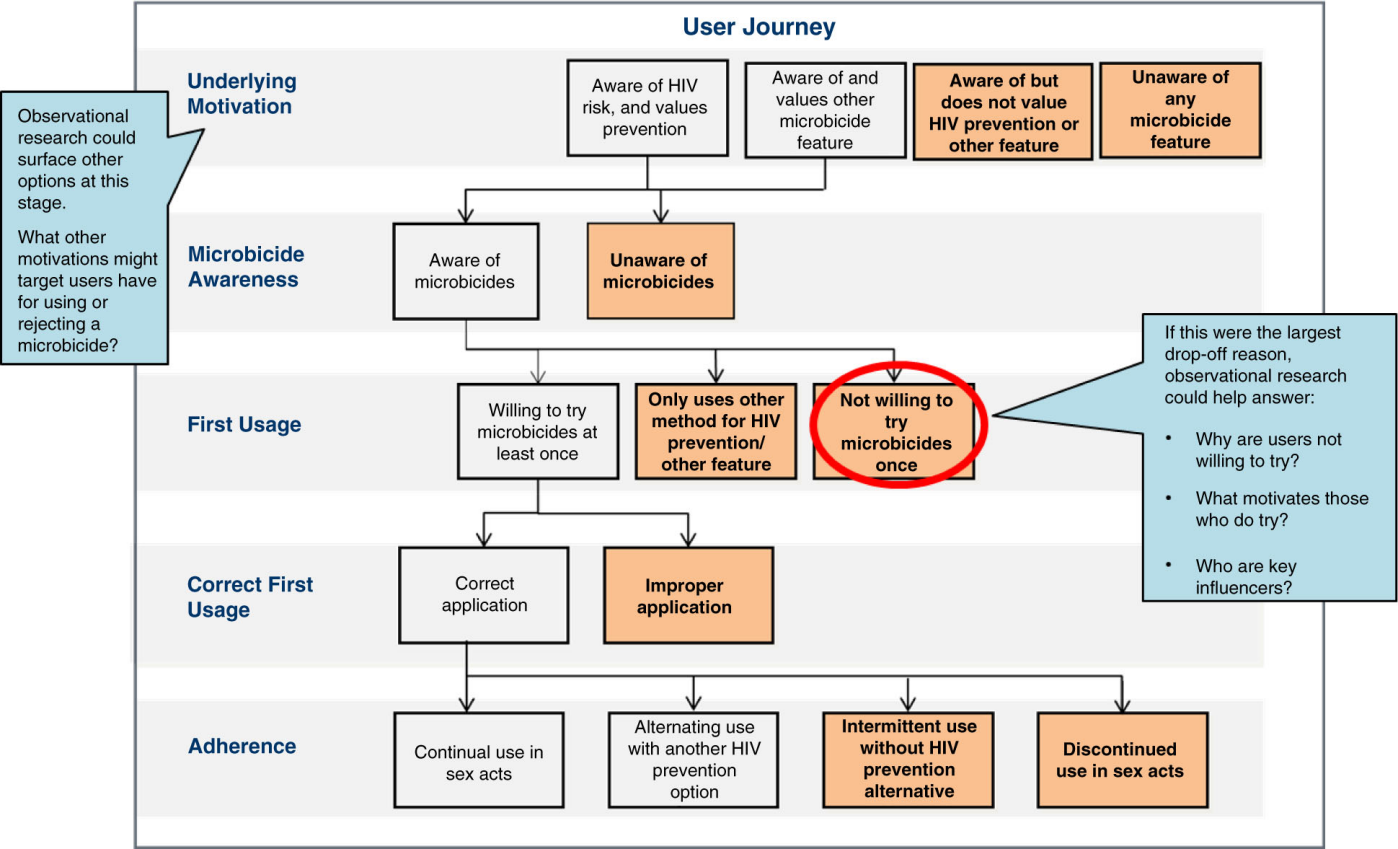
User journey mapping for microbicide user illustrating opportunities to identify potential drop-off points and barriers, drivers and influencers at each stage.

Examining the cascade of care is not a foreign concept for researchers in the HIV field. What is perhaps different in emphasis amongst user-centred private sector firms is the degree of rigour in focusing on the journey from the end-user's perspective rather than the clinical and health system utilization components of a supply-side oriented perspective. By focusing on the end-user experience with microbicides over time, the user journey framework can enable stages to be identified; examined for important barriers, drivers and influencers of microbicide use; and prioritized for further study to ultimately understand and address root causes of low uptake and adherence. Figure 1 illustrates how this iterative user journey mapping process might be used for microbicide users as well as the information it might provide.

### Applying user research to product messaging, positioning and packaging

Synthesis of user research and resulting insights may open possibilities beyond those provided through biomedical trials, behavioural sciences and similar work with a greater focus on the public health interests of disease prevention and drug properties. Bedsider.org, a campaign designed by IDEO with the National Campaign to Prevent Teen and Unwanted Pregnancies, addresses unplanned pregnancy among young women. Through stakeholder research, Bedsider focused on five areas for behavioural change, including awareness and motivational drivers. By offering medically accurate information through a sex-positive brand and addressing a stigmatized topic with humour (versus a clinical, prescriptive tone) and various kinds of media (video, testimonials and other interactions), Bedsider's communication is more engaging and effective for the target audience of women aged 18–29, among whom 7 out of 10 pregnancies are unplanned. To date, over one million users have already visited Bedsider.

While in some cases ‘demedicalizing’ a product may be important, highlighting a product's medical nature can also increase uptake. A psoriasis treatment manufacturer thought potential users were anxious about side effects and marketed their treatment as a beauty product, or ‘just like a cream’. User research conducted by ReD Associates revealed that patients thought it wasn't working and stopped using it. The manufacturer changed the packaging to emphasize potency, and usage increased. With microbicides, user research should similarly play an important role in informing messaging, positioning and packaging.

### Constraints and challenges

In addition to the illustrative techniques outlined above, many other non-traditional methods to understand end-users can be explored for microbicide introduction planning, including co-designing, storyboarding, role plays and drawing as response. Whichever methods are chosen, the following constraints and challenges may arise.

One key issue is how to best test acceptability of a new product when it does not yet exist. Though acceptability assessments are incorporated from phase I to phase III clinical trials, it is critical to gain a more comprehensive understanding of acceptability among the broader (i.e. non-clinical trial participant) population, especially before a completely new class of agents is introduced. One potential workaround is developing an early prototype during the pre-licensure phase and observing how a more diverse population of potential users interacts with this mock product. For example, IDEO developed an insulin starter kit that users could see and touch, enabling researchers to understand how injection instructions were best integrated into the kit, which visuals and text resonated emotionally, and where the kit would be stored at home. By enabling users to physically engage with a prototype and role-play, researchers could better understand how users view the product and how it would fit into users’ lives [28, 30].

Prior to microbicide licensure, lubricants or contraceptive vaginal rings that are already on the market may provide opportunities to conduct user-focused research with products that have commonalities with microbicides. Although motivations for using HIV prevention products are closely linked to risk perception, there are likely to be additional marketing cues that can encourage use, such as product packaging (e.g. colour choice, images, similarities to other product packaging), complexity of instructions or inserts (e.g. terminology, written versus visual directions) and other factors. Conducting this type of acceptability assessment with a broader population can inform the design that is most likely to maximize use and adherence among target demographics.

Another important aspect for user research is to recognize the product promoter's (or promoters’) constraints. These can be investment ceilings, insufficient human resources, or strict regulatory requirements that restrict the ability to act on user insights. It is important to take stock of these constraints early on and to conduct user research with an eye towards producing practical, actionable recommendations. In the case of microbicides, late-stage products using an ARV as the active pharmaceutical ingredient are unlikely to be available for sale over-the-counter, and thus could not be physically placed on the same shelf as other female-focused products like cosmetics, sanitary pads or lubricants. However, early product design prototypes might include packaging that combines condoms and microbicides, emphasizes beauty, promotes sexual pleasure advantages and/or is described by health care service providers in the context of broader issues of women's health and family planning. Instead of messaging only HIV prevention, these approaches might inspire desire for a product, modelled after consumer behaviours (versus patient behaviours).

Lastly, ethical considerations may constrain end-user research that can be done with regard to microbicides in comparison to common consumer products. The examples highlighted in this commentary – for example, contraceptives, diabetes medication and mammography – illustrate that these methods can be successfully and ethically applied to sensitive medical products.

## Conclusions

While biomedical HIV trials and sexual health research utilizes multidisciplinary methods to encourage uptake and adherence in introducing new products, additional user-centred private sector techniques can be integrated outside of the clinical trial context to enhance microbicide introduction planning efforts. In-depth observation, user journey mapping, early prototyping and additional approaches can be employed, as described in the examples above to gain a more comprehensive understanding of the end-user, her environment and the systems with which she interacts. Leveraging such methods will help the field better identify and create opportunities to target users and their key influencers with microbicide messages and positioning that will maximize uptake and adherence.

## References

[1] Baeten JM, Donnell D, Ndase P, Mugo NR, Campbell JD, Wangisi J (2012). Antiretroviral prophylaxis for HIV prevention in heterosexual men and women. N Engl J Med.

[2] Berger RE (2011). Effectiveness and safety of tenofovir gel, an antiretroviral microbicide, for the prevention of HIV infection in women. J Urol.

[3] Grant RM, Lama JR, Anderson PL, McMahan V, Liu AY, Vargas L (2010). Preexposure chemoprophylaxis for HIV prevention in men who have sex with men. N Engl J Med.

[4] Karim SSA, Kashuba AD, Werner L, Karim QA (2011). Drug concentrations after topical and oral antiretroviral pre-exposure prophylaxis: implications for HIV prevention in women. Lancet.

[5] Thigpen MC, Kebaabetswe PM, Paxton LA, Smith DK, Rose CE, Segolodi TM (2012). Antiretroviral preexposure prophylaxis for heterosexual HIV transmission in Botswana. N Engl J Med.

[6] Van Damme L, Corneli A, Ahmed K, Agot K, Lombaard J, Kapiga S (2012). Preexposure prophylaxis for HIV infection among African women. N Engl J Med.

[7] Marrazzo J, Ramjee G, Nair G, Palanee T, Mkhize B, Nakabiito Taljaard M (2013). Pre-exposure prophylaxis for HIV in women: daily oral tenofovir, oral tenofovir/emtricitabine or vaginal tenofovir gel in the VOICE study (MTN 003).

[8] Braunstein S, van de Wijgert J (2005). Preferences and practices related to vaginal lubrication: implications for microbicide acceptability and clinical testing. J Womens Health (Larchmt).

[9] Koblin BA, Andrasik M, Austin J (2013). Preparing for the unexpected: the pivotal role of social and behavioral sciences in trials of biomedical HIV prevention interventions. J Acquir Immune Defic Syndr.

[10] Rausch DM, Grossman CI, Erbelding EJ (2013). Integrating behavioral and biomedical research in HIV interventions: challenges and opportunities. J Acquir Immune Defic Syndr.

[11] Stirratt MJ, Gordon CM (2008). Adherence to biomedical HIV prevention methods: considerations drawn from HIV treatment adherence research. Curr HIV/AIDS Rep.

[12] Tolley EE, Severy LJ (2006). Integrating behavioral and social science research into microbicide clinical trials: challenges and opportunities. Am J Public Health.

[13] Terris-Prestholt F, Hanson K, MacPhail C, Vickerman P, Rees H, Watts C (2013). How much demand for new HIV prevention technologies can we really expect? Results from a discrete choice experiment in South Africa. PLoS One.

[14] Bentley ME, Morrow KM, Fullem A, Chesney MA, Horton SD, Rosenberg Z (2000). Acceptability of a novel vaginal microbicide during a safety trial among low-risk women. Fam Plan Perspect.

[15] Bentley ME, Fullem AM, Tolley EE, Kelly CW, Jogelkar N, Srirak N (2004). Acceptability of a microbicide among women and their partners in a 4-country phase I trial. Am J Public Health.

[16] Greene E, Batona G, Hallad J, Johnson S, Neema S, Tolley EE (2010). Acceptability and adherence of a candidate microbicide gel among high-risk women in Africa and India. Cult Health Sex.

[17] Morrow K, Rosen R, Richter L, Emans A, Forbes A, Day J (2003). The acceptability of an investigational vaginal microbicide, PRO Gel, among women in a phase I clinical trial. J Women’s Health (2002).

[18] Reiff M, Wade C, Chao MT, Kronenberg F, Cushman LF (2008). Health practices and vaginal microbicide acceptability among urban black women. J Womens Health (Larchmt).

[19] Rosen RK, Morrow KM, Carballo-Diéguez A, Mantell JE, Hoffman S, Gai F (2008). Acceptability of tenofovir gel as a vaginal microbicide among women in a phase I trial: a mixed-methods study. J Women’s Health (2002).

[20] Weeks MR, Mosack KE, Abbott M, Sylla LN, Valdes B, Prince M (2004). Microbicide acceptability among high-risk urban U.S. women: experiences and perceptions of sexually transmitted HIV prevention. Sex Transm Dis.

[21] Mngadi K, Maarschalk S, Grobler A, Mansoor L, Frohlich J, Madlala B (2014). Disclosure of microbicide gel use to sexual partners: influence on adherence in the CAPRISA 004 trial. AIDS Behav.

[22] Mansoor L, Karim Q, Werner L, Madlala B, Ngcobo N, Cornman D (2014). Impact of an adherence intervention on the effectiveness of tenofovir gel in the CAPRISA 004 trial. AIDS Behav.

[23] Morrow KM, Fava JL, Rosen RK, Vargas S, Shaw JG, Kojic EM (2013). Designing preclinical perceptibility measures to evaluate topical vaginal gel formulations: relating user perceptions and experiences to formulation properties. AIDS Res Hum Retroviruses [Internet].

[24] Van den Berg JJ, Rosen RK, Bregman DE, Thompson LA, Jensen KM, Kiser PF (2014). “Set it and forget it”: women's perceptions and opinions of long-acting topical vaginal gels. AIDS Behav.

[25] Gafos M, Mzimela M, Sukazi S, Pool R, Montgomery C, Elford J (2010). Intravaginal insertion in KwaZulu-Natal: sexual practices and preferences in the context of microbicide gel use. Cult Health Sex.

[26] Hall P (2011). IDEO takes on the government. Metropolis.

[27] Jahnke M (2009). Innovation through design thinking. Business.

[28] Brown T, Wyatt J (2010). Design thinking for social innovation. Stanford Soc Innovat Rev.

[29] Brown T (2008). Design thinking. Harv Bus Rev.

[30] Buchenau M, Francisco IS, Suri JF Experience prototyping.

[31] Whatmore J (2001). The art of innovation: success through innovation the IDEO way. Long Range Plan.

[32] Suri JF, Howard SG (2006). Going deeper, seeing further: enhancing ethnographic interpretations to reveal more meaningful opportunities for design. J Advert Res.

[33] Madsbjerb C, Rasmussen M (2014). The moment of clarity: using the human sciences to solve your toughest business problems.

[34] Sigall H, Aronson E, Van Hoose T (1970). The cooperative subject: Myth or reality?. J Exp Soc Psychol.

[35] Wang C, Burris MA (1997). Photovoice: concept, methodology, and use for participatory needs assessment. Health Educ Behav.

[36] Wang CC (1999). Photovoice: a participatory action research strategy applied to women's health. J Womens Heal Off Publ Soc Adv Womens Heal Res.

[37] Irwin R (2002). IDEO's design cure: can it fix our sick health-care system?. Metropolis.

[38] Shah A, Thathachari J, Agarwal R, Karamchandani A (2013). A market led, evidence based, approach to rural sanitation [Internet].

